# *MsLHY* is an active regulator of cold resistance in alfalfa (*Medicago sativa* L.)

**DOI:** 10.3389/fpls.2025.1559988

**Published:** 2025-05-01

**Authors:** Jikai Li, Lu Chai, Mei Yang, Hailing Zhang, Chen Shang, Yuxuan Liu, Kailin Qian, Jiuding Sun, Weibo Han, Pan Zhang

**Affiliations:** ^1^ Institute of Grass Research, Heilongjiang Academy of Agricultural Sciences, Harbin, China; ^2^ Department of Grassland Science, College of Animal Science and Technology, Northeast Agricultural University, Harbin, China

**Keywords:** *Medicago sativa* L., late-elongated hypocotyl, low temperature, interacting protein, transgenic alfalfa

## Abstract

Low-temperature stress is a major environmental factor that limits the yield, quality, and geographical distribution of forage crops and restricts the development of the forage industry. As a core component of plant circadian clocks, Late Elongated Hypocotyl (LHY) plays a crucial role in regulating plant rhythms and responses to abiotic stress. However, the molecular mechanism by which *LHY* regulates the cold tolerance of alfalfa has not been reported. In this study, *MsLHY*, which is 2,235 bp in length and encodes 744 amino acids, was isolated from alfalfa. *MsLHY* was highly expressed in roots and stems and was significantly induced by low temperature. Transgenic *MsLHY*-overexpressing (OE) and RNAi alfalfa plants were obtained via *Agrobacterium*-mediated transformation. Under low-temperature stress, OE plants presented reduced reactive oxygen species accumulation and more osmotic regulatory substances, as well as greater antioxidant enzyme activity, to combat cold stress. Conversely, the RNAi plants presented trends opposite those of the OE plants. Furthermore, under cold stress, the overexpression of *MsLHY* upregulated the expression of the cold-responsive genes *MsICE1*, *MsCBF1*, *MsCOR15A*, and *MsCML10*, as well as the expression of the antioxidant-synthesizing genes *MsSOD1* and *MsCAT1*, thereby increasing the cold tolerance of transgenic alfalfa. These results suggest that *MsLHY* plays an important role in increasing the cold tolerance of alfalfa.

## 1 Introduction

Abiotic stress, especially cold stress, has major effects on plant growth and development (Mickelbart et al., 2015; Ritonga and Chen, 2020), and the agricultural economy suffers losses of hundreds of billions of yuan every year due to low temperatures (Li and Christersson, 1992). Low-temperature stress results in cold damage at low temperatures above 0°C and freezing damage at low temperatures below 0°C (Chen et al., 2022). After sensing low-temperature signals, plants resist low-temperature stress through a series of molecular mechanisms and physiological regulation. C-repeat-binding factors (CBFs), inducers of CBF expression (ICEs) and cold-regulated genes (CORs) play key roles in plant cold adaptation (Lee and Thomashow, 2012; Ding et al., 2019). These genes form the ICE-CBF-COR pathway, which alleviates plant damage caused by low-temperature stress (Thomashow, 1999; Shu et al., 2017; Wang et al., 2017; Guo et al., 2019), and the active elements of the ICE-CBF-COR pathway have been identified in rice and other crop species (Bremer et al., 2017). In addition, the activation of protective enzymes and metabolites related to cold resistance; the synthesis of protective proteins and metabolism-related complexes; and the maintenance of a new energy balance, metabolite balance and redox balance are crucial for increasing low-temperature tolerance in plants (Murata et al., 2007; Grabelnych et al., 2014; Shi et al., 2018; Ambroise et al., 2020).

Late Elongated Hypocotyl (LHY) is a key circadian clock gene in *Arabidopsis thaliana* that encodes a protein belonging to the MYB family of transcription factors that play a central role in regulating the circadian rhythm of plants, affecting their growth, flowering time, and response to environmental changes (Mi-Jeong et al., 2016; Adams et al., 2018). The LHY protein contains several functional domains, including the SANT, homeodomain-like, Myb, and DNA-binding domains. These domains give LHY the ability to bind to DNA, thereby regulating the expression of specific genes (Atamian and Harmer, 2016). LHY, together with Circadian Clock Associated 1 (CCA1) and Timing Of Cab 1 (TOC1), constitute the core oscillators of the plant biological clock (Hung et al., 2019; Su et al., 2021). The expression of Pseudo-Response Regulator (PRR) family genes is inhibited by LHY and CCA1, and these genes in turn negatively regulate the expression of CCA1 and LHY, forming a complex regulatory network (Farre and Liu, 2013). The *LHY* gene also accelerates flowering in plants under continuous light conditions by promoting the expression of the Flowering Locus T (FT) gene (Liu et al., 2024).

In addition, the *LHY* gene plays an important role in the abiotic stress response. This gene influences drought tolerance in plants by regulating the response of the plant hormone abscisic acid (ABA) (Adams et al., 2018). Studies have shown that under drought conditions, the expression of the *LHY* gene increases, directly inhibiting the target genes of multiple ABA signaling pathways and thus affecting the drought response of plants (Wang et al., 2020). Moreover, the *LHY* gene is associated with water use efficiency (WUE) in plants, regulating water retention and use by influencing ABA biosynthesis and signaling (Xu et al., 2022). Studies have also shown that LHY transcription factors are involved in the regulation of the plant response to low temperature, especially during vernalization. LHY together with CCA1 activates VIN3 transcription through the vernalization reaction cis-element, which is a key step in the acquisition of long-term cold-mediated flowering ability (Kyung et al., 2022). The expression of the *LHY* gene also changed under low-temperature stress, and the cold-induced expression of the *DREB1* gene in the *cca1 lhy* double mutant *A. thaliana* significantly decreased (Dong et al., 2011). EEs are present in the promoter region of DREB1 and many cold-induced genes, and CCA1 LHY can bind EEs to inhibit the expression of evening genes (Harmer and Kay, 2005). These findings reveal the regulatory mechanism of the *LHY* gene during plant adaptation to low-temperature environments and provide an important molecular basis for further research and improvement of plant cold tolerance.

Alfalfa (*Medicago sativa* L.), known as the “king of grass”, is an excellent perennial grass of the Leguminosae family, with a high protein content and high nutritional value (Annicchiarico et al., 2015). Its strong adaptability in various soil and climate conditions, is an indispensable component of animal husbandry (Wang et al., 2024). In northern China, winters are cold, and extremely low temperatures occur frequently, during late spring and early fall causing major losses in the growth and production of alfalfa; thus, low temperatures are among the major environmental factors restricting the industrialization of alfalfa in northern China (Liu et al., 2006). Therefore, studying the physiological and molecular response mechanisms of alfalfa under low-temperature stress, increasing its adaptability and productivity in cold areas, reducing economic losses caused by low temperature, promoting the sustainable development of animal husbandry, and providing a scientific basis for breeding cold-resistant varieties are highly important.

The LHY-like protein associated with cold stress was identified in our previous transcriptomic data, but the function of *LHY* and its role in cold tolerance have not been fully elucidated. In this study, we cloned *MsLHY* and examined its relative expression level under abiotic stress to determine how it is expressed under abiotic stress and found that *MsLHY* is strongly induced by low temperature. Compared with RNAi plants, *MsLHY-*overexpressing transgenic alfalfa presented increased cold tolerance at 4°C and -5°C. Similarly, the expression of cold stress response genes in overexpressing plants was also significantly induced to increase the cold tolerance of alfalfa. In this study, the function and regulatory mechanism of the *LHY* gene in the cold tolerance of alfalfa was explored to provide a knowledge basis for the breeding and genetic improvement of cold-tolerant varieties of alfalfa.

## 2 Materials and methods

### 2.1 Plant materials and stress treatments

Alfalfa (*M. sativa* L. cv. Zhongmu No. 1) seeds were treated with 75% ethanol solution for 60 s, followed by 10% NaClO solution treatment for 10 min, washed with sterile water 5 times and placed on Petri dishes with wet filter paper. After 5 days of germination, healthy seedlings with uniform growth were selected and moved to a cavity dish filled with soil and vermiculite at a ratio of 1:1, placed in an incubator under a 16/8 h light/dark photoperiod (25/20°C) with 60% relative humidity, and watered with alfalfa optimized nutrient solution every 2 days (Zhang, 2014). When reaching the five-leaf stage, the seedlings were subjected to osmotic stress to simulate drought (20% PEG-6000), salt (150 mM NaCl), saline–alkaline (150 mM NaHCO_3_) or low-temperature (4°C) conditions. Samples were collected from above and below ground at 0 h, 2 h, 4 h, 8 h, 12 h, 24 h and 48 h, with 3 biological replicates per treatment. The samples were stored in a labeled centrifuge tube, rapidly frozen in liquid nitrogen and stored at -80°C.

### 2.2 Full-length CDS cloning of *MsLHY*

The total RNA of alfalfa was extracted using an RNA extraction kit (Vazyme, Nanjing, China) according to the manufacturer’s instructions, and the first strand of cDNA was synthesized according to the manufacturer’s instructions (Vazyme, Nanjing, China). Specific gene cloning primers (**Supplementary Table S1**) were designed using the reference sequence of the genome (AES82836.2) of *Medicago truncatula*, and 50 μL polymerase chain reaction (PCR) was used to amplify the gene fragment using alfalfa cDNA as a template. The PCR amplification program was as follows: 95°C for 3 min; 35 cycles of 95°C for 10 s, 60°C for 20 s, and 72°C for 40 s; and 72°C for 5 min (Vazyme, Nanjing, China). The fragment length was verified via agarose gel electrophoresis. The DNA fragments were purified using a product purification kit (Vazyme, Nanjing, China), the purified product was ligated into the pCE2 TA/Blunt-Zero Cloning vector using a 5-min TA/Blunt-Zero Cloning Kit (Vazyme, Nanjing, China), and the recombinant product was introduced into *E. coli* DH5α (Weidi, Shanghai, China).

### 2.3 Bioinformatic analysis of *MsLHY*

The amino acid sequence was obtained via transcriptomic approaches. The *MsLHY* sequence was obtained from SnapGene. The homologous sequences of MsLHY were identified from NCBI (https://www.ncbi.nlm.nih.gov/), multiple sequence comparisons were performed using DNAMAN 6.0.3.99, a phylogenetic tree was constructed using MEGA 7.0, and the phylogenetic tree was visualized using Evolview (https://www.evolgenius.info/evolview/#/treeview). Domains in the amino acid sequence encoded by MsLHY was predicted using SMART (http://smart.embl.de/smart/set_mode.cgi?GENOMIC=1), and the physicochemical properties of the MsLHY protein were analyzed using ExPASy (https://web.expasy.org/protparam/). PredictProtein (https://predictprotein.org/) and SWISS-MODEL (https://swissmodel.expasy.org/) were used to predict the secondary structure of the MsLHY protein, and a tertiary structure model was constructed. NetPhos 3.1 (https://services.healthtech.dtu.dk/services/NetPhos-3.1/), ProtScale (https://web.expasy.org/protscale/), TMHMM Server v.2.0 (http://www.cbs.dtu.dk/services/TMHMM-2.0/) and SignalP 4.1 (http://www.cbs.dtu.dk/services/SignalP-4.1/) were used to analyze and predict the phosphorylation sites, hydrophilicity, transmembrane domains and signal peptides of the MsLHY protein.

### 2.4 Interacting protein prediction

After the MsLHY protein sequence was imported into the STRING (https://cn.string-db.org/) database, the proteins that may interact with MsLHY were analyzed and predicted.

### 2.5 Gene expression analysis

Quantitative real-time PCR (qRT–PCR) primers (**Supplementary Table S1**) for *MsLHY* were designed using NCBI Primer BLAST (http://www.ncbi.nlm.nih.gov/tools/primer-blast/). The alfalfa *GAPDH* gene was used as the internal housekeeping gene for qRT–PCR (q225MX-400, Kubotech, China) to determine the expression levels and tissue-specific expression levels of *MsLHY* under different stresses. The qRT–PCR amplification program was as follows: 95°C for 30 s; 40 cycles of 95°C for 10 s, 60°C for 30 s, and 95°C for 15 s; 60°C for 1 min; and 95°C for 15 s. The 2^−ΔΔCT^ comparative method was used to determine how each gene was expressed (Livak and Schmittgen, 2001).

### 2.6 Generation of *MsLHY* transgenic plants

The PEG100 vector (Biorun, Wuhan, China) was digested with *BamH*I and *Xba*I, and the target gene was inserted into the linearized vector to obtain the PEG100-MsLHY-OE recombinant overexpression vector. A PEG100-MsLHY-RNAi recombinant RNAi vector was obtained by inserting 200 bp *MsLHY* gene-specific sequences and loop structure into the PEG100 vector cut by the *BamH*I and *Xba*I enzymes by Golden Gate method. After two recombinant vectors were transformed into the *Agrobacterium tumefaciens* strain EHA105 (Weidi, Shanghai, China), alfalfa was transformed via the *Agrobacterium*-mediated leaf disc method (Ke et al., 1998). Since the PEG100 vector has a Bar resistance gene, 1 mg·L^-1^ Basta was used to screen for transformed plants. The transformants were transplanted into the soil, and the leaves of the screened plants were assessed using Bar test paper (Biorun, Wuhan, China). RNA was extracted and reverse-transcribed into cDNA. PCR detection was subsequently performed using Bar gene-specific primers (**Supplementary Table S1**). Specific qPCR primers for *MsLHY* were designed, and the relative expression level of *MsLHY* in the transgenic plants was determined with the alfalfa *GAPDH* gene as the internal housekeeping gene (**Supplementary Table S1**).

### 2.7 Cold treatment and determination of the physiological indices of the transgenic plants

WT and transgenic alfalfa were propagated via the asexual cutting propagation method; three plants were planted in a pot of vermiculite and nutrient soil (1:1), cultivated under a 16/8 h light/dark photoperiod (25/20°C) with 60% relative humidity, and watered with alfalfa optimized nutrient solution every 3 days. After five weeks, the pots were placed in an incubator at 4°C and -5°C with 16/8 h day/night and 60% humidity for cold resistance determination. To mimick temperature drop in nature, the incubator was subjected to gradient cooling at 1°C/h. After treatment at 4°C for 7 days and -5°C for 8 h, samples were taken from the aboveground parts of the plants at the same time point in the photoperiod, with 3 biological replicates per treatment. The plants were stored in a marked centrifuge tube, rapidly frozen in liquid nitrogen and stored at -80°C. The relative conductivity was determined according to the methods of Song et al. (2006) with some modifications. Leaves (0.1 g) were incubated in 10 mL of deionized water at 4°C for 12 h. The relative conductivity (C_1_) of the incubated solution was determined. The sample was then boiled for 15 min, and the conductivity (C_2_) of the solution was again determined. The relative conductivity was calculated as (C_1_/C_2_) × 100%. Superoxide dismutase (SOD) activity, peroxidase (POD) activity, catalase (CAT) activity, soluble protein (SP) content, soluble sugar (SS) content, hydrogen peroxide (H_2_O_2_) content, superoxide anion (O_2_^-^) content, proline (Pro) content and malondialdehyde (MDA) content were measured using a kit (Comin, Suzhou, China) using living tissue.

### 2.8 Analysis of expression of stress response genes

The expression of six genes involved in the low-temperature stress response pathway in WT and transgenic alfalfa was determined via qRT–PCR to understand how *MsLHY* responds to low-temperature stress at the gene level. *MsICE1*, *MsCBF1* and *MsCOR15A* are the key genes of the cold stress pathway. *MsSOD1* and *MsCAT* encode SOD and CAT, respectively. The *MsCML10* gene encodes a member in cold-induced Ca^2+^ signaling and plays a key role in regulating plant cold tolerance (Yu et al., 2022). The *GAPDH* gene is an internal housekeeping gene. The sequences of the primers used in this study are listed in **Supplementary Table S1**.

### 2.9 Statistical analyses

Statistical analysis of the results was performed via one-way analysis of variance (ANOVA) using SPSS 19, and Duncan’s multiple range test was used to identify significant differences between groups. The means were considered significantly different at *p* < 0.05.

## 3 Results

### 3.1 Characterization and Isolation of *MsLHY*

**Figure 1:**
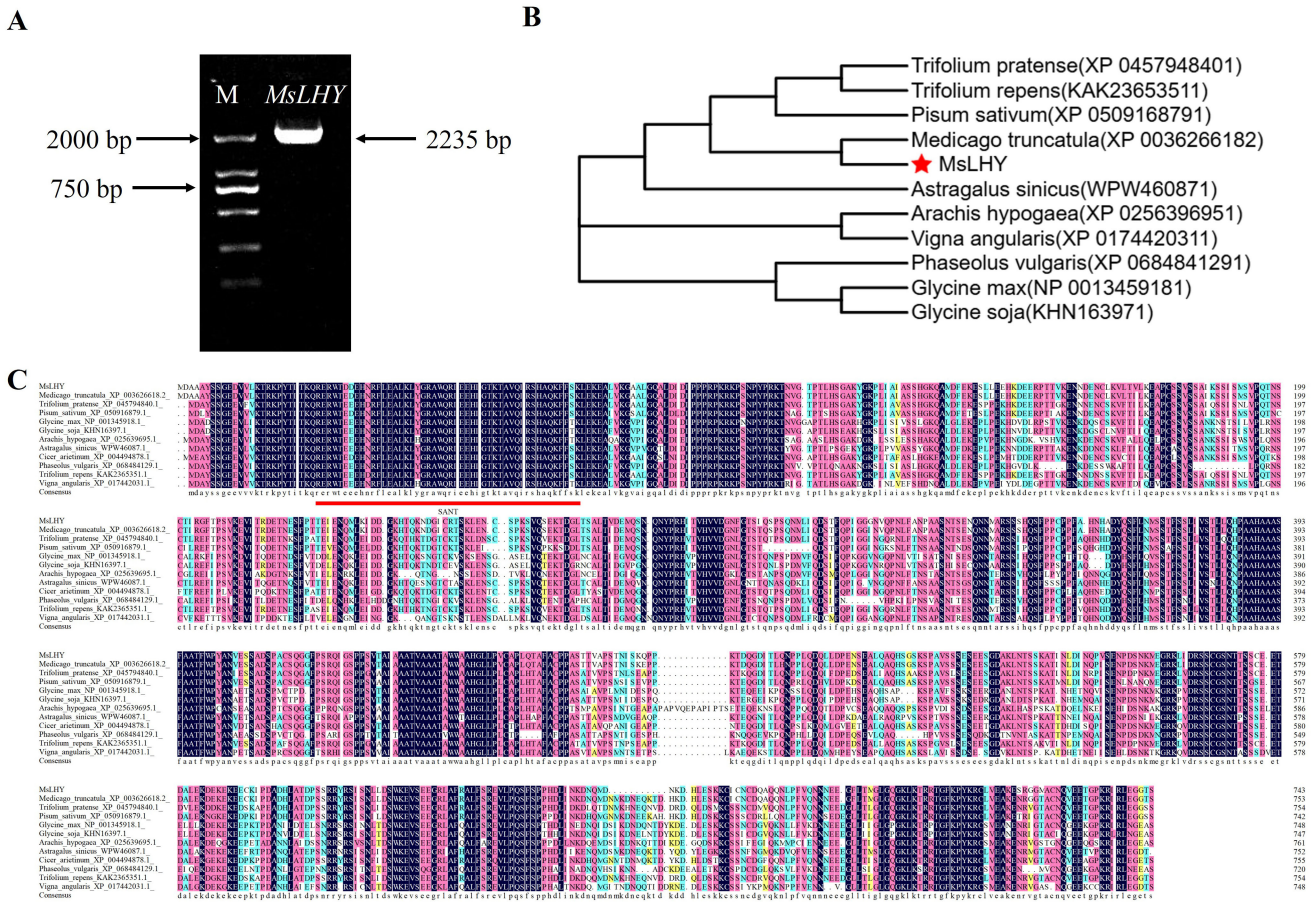
Gene isolation, multiple sequence alignment and phylogenetic tree of MsLHY. **(A)** Cloning of *MsLHY*. **(B)** Phylogenetic tree of MsLHY. **(C)** Amino acid sequence alignment.

We previously identified LHY-like proteins in a transcriptomic study. The full-length cDNA sequence of *M. sativa* L. cv. Zhongmu No. 1 was used as a template, and the *MsLHY* gene was subsequently cloned using a specific primer designed with the reference sequence of *M. truncatula* (AES82836.2). Sequence analysis revealed that MsLHY has an open reading frame of 2,235 bp (**Figure 1A**) encoding a protein of 744 amino acids with the molecular formula C_3501_H_5565_N_1015_O_1155_S_29_. The predicted molecular weight is 81.286 kDa, and the theoretical isoelectric point (pI) is 6.03. SMART domain analysis revealed the SANT domain at amino acids 25-73, indicating that MsLHY is a member of the Myb/SANT family (**Supplementary Figure S1A**). The secondary structure of the MsLHY protein consists of 17.65% α-helices, 4.41% β-turn and 57.94% random coils. The content of serine (Ser) was the highest in terms of amino acid composition, accounting for 11.2% (**Supplementary Figure S1B**). The alanine (Ala) content, 8.3%, was second only to the serine content. Ninety-one amino acid residues were negatively charged, and 81 were positively charged. The instability index of the MsLHY protein was 56.68 (greater than 40), indicating that the protein could not exist stably *in vitro* and was unstable. The fat solubility index was 64.45.

The physicochemical property analysis revealed 94 predicted phosphorylation sites for the MsLHY protein, including 62 serine phosphorylation sites (serine), 2 tyrosine phosphorylation sites (tyrosine), and 30 threonine phosphorylation sites (threonine) (**Supplementary Figure S2A**). The average hydrophilic value of MsLHY is -0.654, indicating that MsLHY is a hydrophilic protein (**Supplementary Figure S2B**). The MsLHY protein has no transmembrane domains (**Supplementary Figure S2C**), no signal peptide splicing sites, and no signal peptide sequences; it is a nonsecretory protein, and it cannot transport proteins (**Supplementary Figure S2D**).

Multiple sequence comparisons of amino acids performed using DNAMAN revealed that the sequences of *M. truncatula* (XP_003626618.2)*, Trifolium pratense (*XP_045794840.1)*, Trifolium repens* (KAK2365351.1)*, Pisum sativum* (XP_050916879.1)*, Cicer arietinum* (XP_004494878.*1), Astragalus sinicus* (WPW46087.1)*, Glycine soja* (KHN16397.1)*, Glycine max* (NP_001345918.1)*, Phaseolus vulgaris* (XP_068484129.1), and *Arachis hypogaea* (XP_025639695.1) presented 97.35%, 85.77%, 85.64%, 83.87%, 82.85%, 76.96%, 72.88%, 71.73%, 67.60%, and 67.23% homology, respectively, with the SANT domains (**Figure 1C**). Phylogenetic trees were constructed, revealing that the MsLHY protein has high homology with MtLHY, which has the closest genetic relationship (**Figure 1B**).

### 3.2 Prediction of MsLHY-interacting proteins

**Figure 2:**
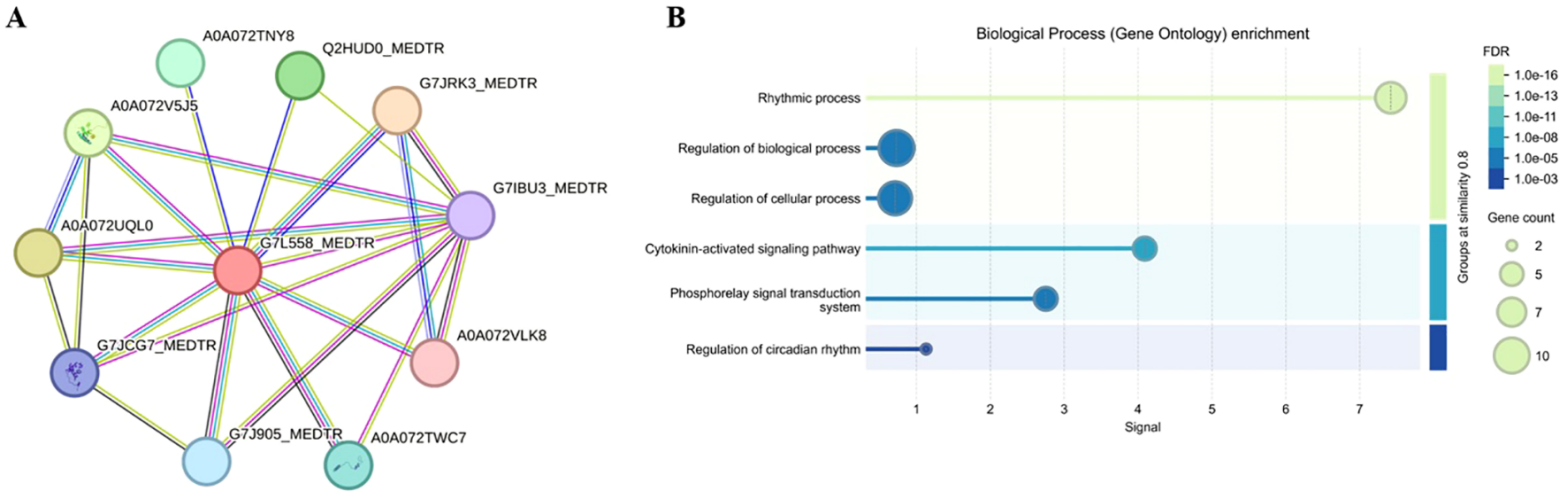
Prediction of MsLHY-interacting proteins using the STRING database. **(A)** Interacting proteins. **(B)** Biological process.

The proteins that may interact with MsLHY were preliminarily predicted using the STRING database (**Figure 2A**). A total of 10 possible interacting proteins were predicted, of which 8 were enriched in the rhythmic process, 2 were enriched in the circadian rhythm, 9 were enriched in cellular processes, 10 were enriched in biological processes, 5 were enriched in the phospholipid signal transduction system, and 5 were enriched in the cytokine-activated signaling pathway (**Figure 2B**). According to our results, the interaction between PRR (G7JRK3_MEDTR) and MsLHY (G7L558_MEDTR) is the most likely interaction.

### 3.3 Expression patterns of *MsLHY*

**Figure 3:**
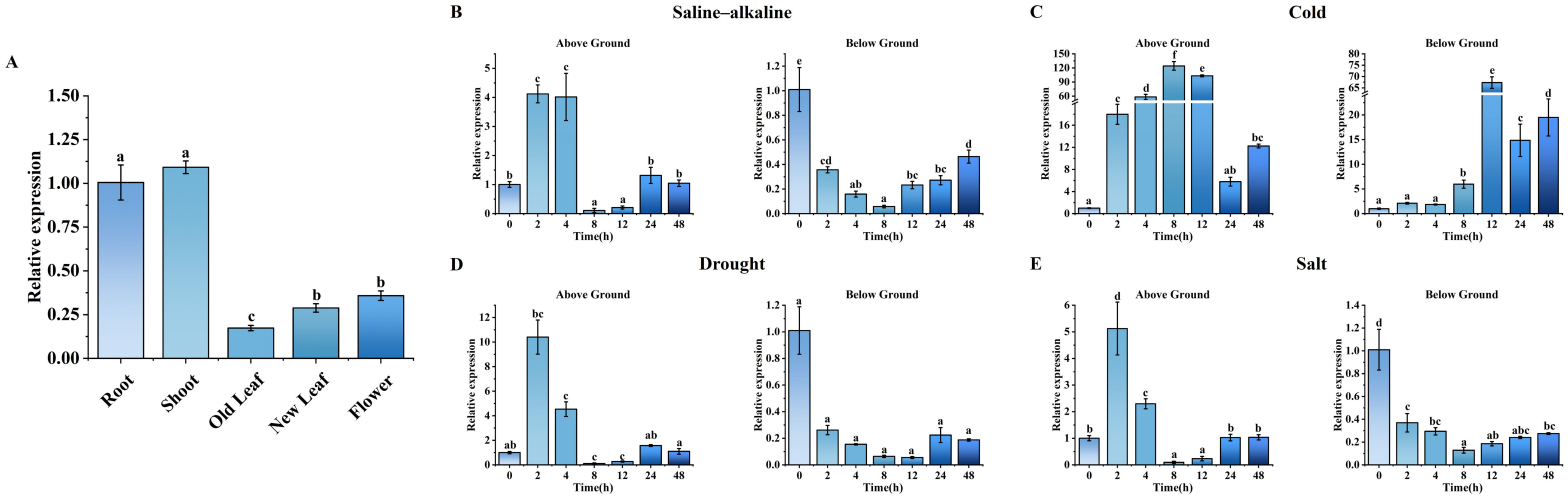
**(A)** qRT–PCR analysis of *MsLHY* expression levels in different alfalfa tissues (root, shoot, old leaf, new leaf and flower tissues). **(B)** Saline–alkaline solution (150 mM NaHCO_3_). **(C)** Cold (4°C). **(D)** Drought (20% PEG-6000). **(E)** Salt (150 mM NaCl). The error bars represent the means ± SDs (n = 3) from three independent biological replicates. Different letters indicate statistically significant differences (Duncan’s tests, p < 0.05).

The relative expression level of *MsLHY* in different alfalfa tissues was the highest in stems and the lowest in old leaves (**Figure 3A**). Treatment with different abiotic stresses revealed that *MsLHY* could be induced by salt, saline–alkaline conditions, drought and low temperature. Compared with that before treatment, after saline–alkaline conditions, drought and salt stress, the relative expression level of *MsLHY* increased first and then decreased in the above-ground, and the expression level recovered to the level before treatment at 48 h (**Figures 3B, D, E**). The relative expression of underground parts decreased overall. However, after low-temperature stress, *MsLHY* was significantly induced in the aboveground parts, and its relative expression level significantly increased within 12 h, reaching 124 times greater than that before treatment at 8 h. The relative expression level of the underground parts reached a maximum at 12 h, i.e., 67 times greater than that before treatment (**Figure 3C**).

### 3.4 Overexpression of *MsLHY* increases cold tolerance in transgenic alfalfa

**Figure 4:**
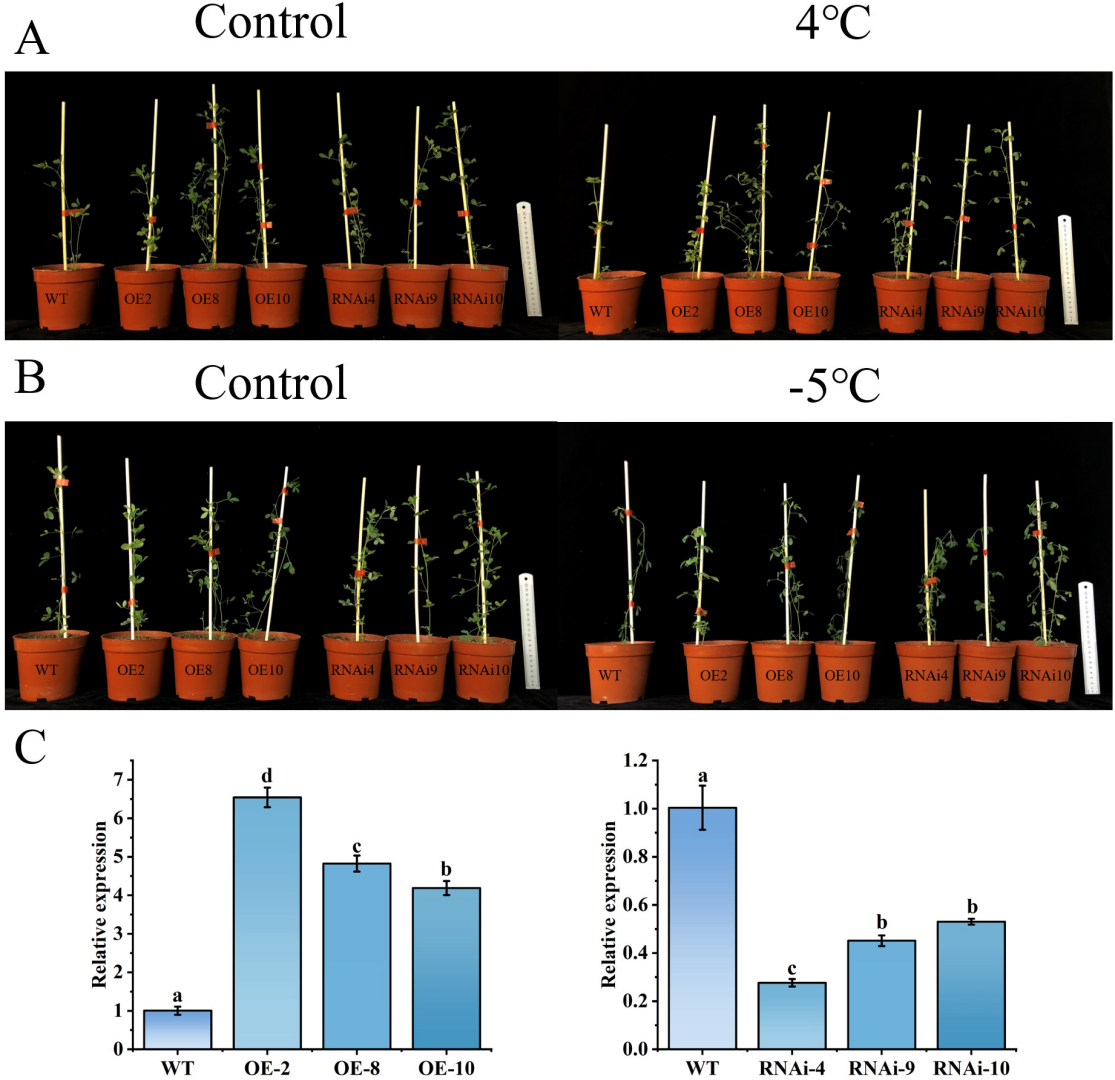
qRT–PCR analysis of the relative expression level of *MsLHY* in transgenic alfalfa and its phenotype under control conditions and low-temperature stress. **(A)** Phenotype of transgenic alfalfa treated at 4°C for 7 days. **(B)** Phenotype of transgenic alfalfa treated at -5°C for 8 hours. **(C)** Identification of transgenic alfalfa via qRT–PCR. The error bars represent the means ± SDs (n = 3) from three independent biological replicates. Different letters indicate statistically significant differences (Duncan’s tests, p < 0.05).

In this study, alfalfa transgenic plants were successfully obtained via the leaf disc method (**Supplementary Figure S3**). Ten plants positive for overexpression (OE) or RNA interference (RNAi) were screened using Bar test paper and RT–PCR, respectively (**Supplementary Figure S4**). The expression level of the *MsLHY* gene was further quantitatively analyzed via qRT–PCR, and three plants were selected on the basis of the expression level for follow-up experiments (**Figure 4C**). At 4°C, there was no difference in plant phenotype, but at -5°C, the leaves of the RNAi-treated and WT plants wilted, and the OE plants grow normally expect for OE-10, which also showed wilty phenotype, OE-10 showed the lowest over-expression which may not be sufficient to lead to significant phenotype change. Measurement of the relative conductivity of the plants after low-temperature stress revealed that the OE plants presented less ion leakage than the WT and RNAi plants did (**Supplementary Figure S5**). These results revealed that the overexpression of *MsLHY* increased the cold tolerance of alfalfa (**Figures 4A, B**).

### 3.5 Physiological changes in transgenic alfalfa under low-temperature stress

**Figure 5:**
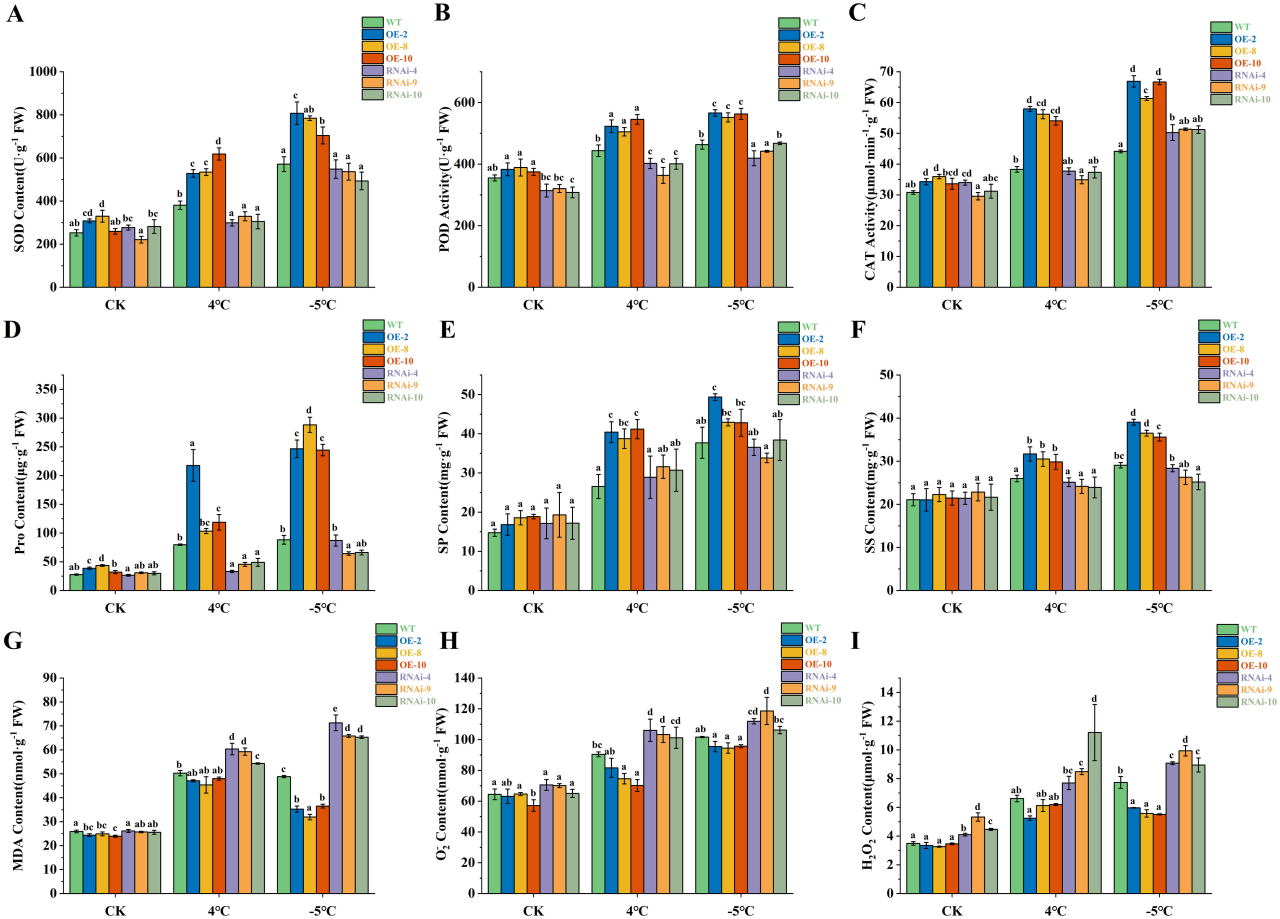
Physiological indices of the control and transgenic plants under 4°C and -5°C stress conditions. The measured indices included the levels of **(A)** SOD, **(B)** POD, **(C)** CAT, **(D)** Pro, **(E)** SP, **(F)** SS, **(G)** MDA, **(H)** O_2_^-^ and **(I)** H_2_O_2_. The error bars represent the means ± SDs (n = 3) from three independent biological replicates. Different letters indicate statistically significant differences (Duncan’s tests, p < 0.05).

To better verify the function of *MsLHY* at low temperatures, we measured the physiological indices of the transgenic alfalfa before and after low-temperature stress. The activities of three antioxidant enzymes (SOD, POD, and CAT) in the OE plants were significantly greater than those in the WT and RNAi plants after low-temperature stress (p<0.05) (**Figures 5A–C**). The CAT activity of the RNAi plants was greater than that of the WT plants after treatment at -5°C and lower than that of the WT plants under other conditions. After low-temperature stress, H_2_O_2_ and O_2_^-^ accumulated in the plants, the content of these molecules in the OE plants was lower than that in the WT plants was, and the content in the RNAi plants was greater than that in the WT plants. In contrast, the content in the OE plants at -5°C was significantly lower than that in the WT plants (p<0.05), indicating that the overexpression of *MsLHY* reduces the accumulation of reactive oxygen species (ROS) (**Figures 5H, I**). The MDA content significantly differed between the OE and RNAi plants and the WT plants at -5°C, and the MDA content in the RNAi plants was significantly greater than that in the WT and OE plants (p<0.05) (**Figure 5G**). Pro plays an important role as an osmotic regulator under low-temperature stress. With the exception of OE-8, the Pro content of the OE plants was significantly greater than that of the WT and RNAi plants (p<0.05) (**Figure 5D**). Under normal conditions, there was no significant difference in the SP and SS contents of all the plants, but OE and RNAi increased these contents to varying degrees after low-temperature treatment. The SP and SS contents of the OE plants were significantly greater than those of the WT and RNAi plants (p<0.05), and there was no significant difference in the SP and SS contents between the RNAi and WT plants (**Figures 5E, F**). In conclusion, the overexpression of *MsLHY* can improve the cold tolerance of alfalfa at the physiological level by increasing the activity of antioxidant enzymes, clearing ROS, and increasing the amount of osmoregulatory substances.

### 3.6 Expression analysis of cold stress-related genes in transgenic alfalfa

**Figure 6:**
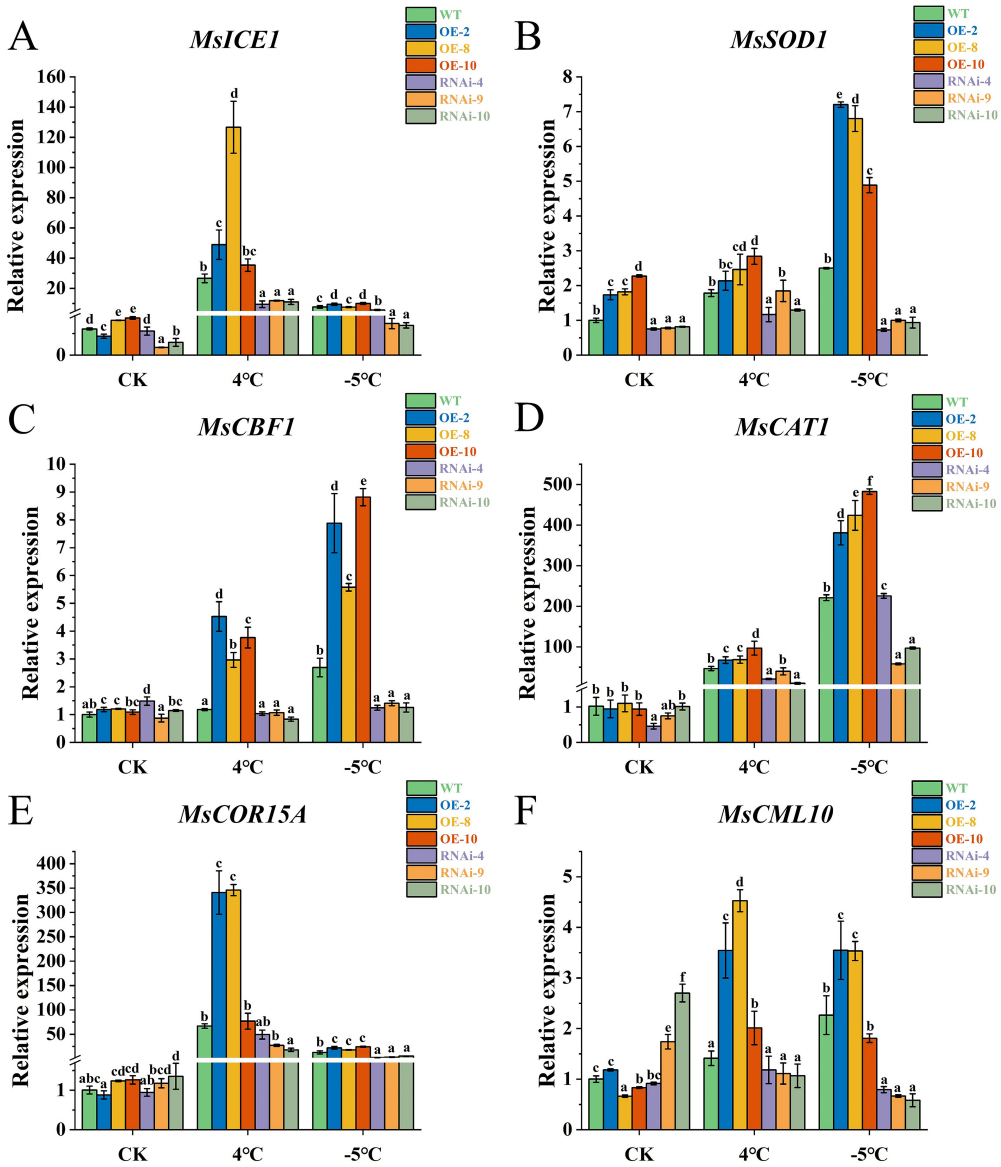
Expression of six related genes in transgenic alfalfa after low-temperature stress. **(A)** *MsICE1*, **(B)** *MsSOD1*, **(C)** *MsCBF1*, **(D)** *MsCAT1*, **(E)** *MsCOR15A* and **(F)** *MsCML10*. The error bars represent the means ± SDs (n = 3) from three independent biological replicates. Different letters indicate statistically significant differences (Duncan’s tests, p < 0.05).

The expression patterns of cold stress response genes and antioxidase synthetic genes in alfalfa were analyzed via qRT–PCR at low temperatures. The results revealed that low temperature significantly increased the expression levels of *MsICE1*, *MsCBF1* and *MsCOR15A* in OE-2 and OE-8 (p<0.05), whereas the expression of only *MsCBF1* was significantly increased in OE-10 (p<0.05) (**Figures 6A, C, E**). Under -5°C stress, the expression levels of *MsSOD1* and *MsCAT1* were significantly increased in the OE plants (p<0.05) but significantly decreased in the RNAi plants (p<0.05) (**Figures 6B, D**). Interestingly, *MsCAT1* expression in RNAi-4 was significantly greater than that in the WT under -5°C stress. Under normal conditions, the expression of *MsCML10* in the RNAi plants was greater than that in the OE plants, but after cold stress, the expression of *MsCML10* in the OE plants was significantly greater than that in the RNAi plants (p<0.05) (**Figure 6F**).

## 4 Discussion

Temperature is the main environmental factor that determines the geographical distribution of plants and affects their growth and production (Ritonga and Chen, 2020). To cope with cold stress, plants have evolved a series of mechanisms to adapt to cold environments, such as adjusting metabolic processes (Theocharis et al., 2012), changing gene expression patterns (Zhu et al., 2007), synthesizing osmoregulatory substances (Dirk et al., 2020), and epigenetic regulation (Liu et al., 2018; Ding et al., 2020). Like those of the tobacco and *Arabidopsis* LHY proteins, the N-terminus of MsLHY also has a conserved SANT domain that is a member of the Myb/SANT family (Yon et al., 2012) (**Supplementary Figure S1**). The expression level of *MsLHY* was the highest in the stems and the lowest in old leaves, suggesting that *MsLHY* may play a regulatory role in plant growth and development (**Figure 3**). Ten interacting proteins enriched in rhythmic processes, cellular processes, biological processes, the phospholipid signal transduction system, and the cytokinin-activated signaling pathway were predicted using the String database (**Figure 2**). G7JRK3_MEDTR may interact with MsLHY (G7L558_MEDTR). G7JRK3_MEDTR is a PRR, and in *A. thaliana*, the interaction between TOC1 (also known as PRR1) and CCA1/LHY is reciprocal. CCA1/LHY can bind to the promoter region of TOC1 and inhibit its expression, and TOC1 can indirectly promote the expression of CCA1/LHY by binding the Constans, Constans-like and TOC1 (CCT) domains to the promoters of CCA1/LHY (Alabadi et al., 2001; Mizoguchi et al., 2002; Gendron et al., 2012).

The *LHY* gene plays an important synergistic role in the abiotic stress process (Wang et al., 2021; Wei et al., 2022) and can induce expression under cold (Nakamichi et al., 2010; Seo et al., 2012), heat (Blair et al., 2019), drought (Wang et al., 2021), salt (Li et al., 2024) and hormone (Zheng et al., 2006) treatments, but the expression of *MsLHY* in alfalfa under stress has not been studied. In this study, *MsLHY* was induced by drought, salt and saline–alkaline conditions and was significantly upregulated under cold stress, which was consistent with the expression of *OsLHY* (Xu et al., 2016) and *FmLHY* (Cao, 2024) after low-temperature stress. These results suggest that *MsLHY* can function at low temperatures. Low temperatures can cause damage to plant cell membranes (Aghdam et al., 2019). Through the *Agrobacterium*-mediated method, we obtained OE and RNAi transgenic alfalfa (**Supplementary Figure S3**). Under cold stress, the growth of the RNAi plants was inhibited, and the relative conductivity of the RNAi plants was greater than that of the WT plants (**Figure 2**), indicating that the cold tolerance of alfalfa was reduced due to the inhibition of *MsLHY*, which was similar to the results of related studies. For example, the cold tolerance of tea plants is reduced by the inhibition of *CsLHY* (Wu et al., 2024). Studies have shown that when *CsLHY* is inhibited, the cold tolerance of *Camellia sinensis* is reduced. SgRVE6 is a transcription factor encoding LHY-CCA1-Like, which can be rapidly upregulated under cold stress, affecting the expression of circadian clock genes and improving the cold tolerance of tobacco (Chen et al., 2020).

Low temperature can lead to the accumulation of ROS in plant cells, and the contents of MDA, H_2_O_2_ and O_2_^-^ reflect the degree of damage caused by low temperature to plants to a certain extent. The results revealed that the increase in the MDA content in cold-resistant sorghum was less than that in cold-sensitive materials (Shi et al., 2015) and that a decrease in the MDA and H_2_O_2_ contents improved the cold tolerance of *M. sativa* (Ye et al., 2024). Plants also remove ROS by increasing the activity of antioxidant enzymes such as SOD and CAT, thereby improving cold resistance (Chen et al., 2014; Wang, 2023). We determined the physiological indices of alfalfa after low-temperature stress and found that the activities of SOD, POD and CAT in OE plants were significantly greater than those in WT plants, whereas the contents of MDA, H_2_O_2_ and O_2_^-^ in RNAi plants were significantly greater than those in WT and OE plants. The enhanced CAT and SOD activities in OE plants were supported by their greater expression levels in plants (**Figures 6B, D**). These results suggest that the overexpression of *MsLHY* improves the ability of alfalfa to scavenge ROS and reduces the accumulation of reactive oxygen species. Consistent with findings in *Arabidopsis*, when the expression of the *LHY* gene is inhibited, the activity of antioxidant enzymes is reduced, increasing the vulnerability of plants to oxidative damage caused by low-temperature stress (Lai et al., 2012). The accumulation of ROS can also lead to an increase in cell membrane permeability, whereas Pro, SS and SP contents can increase after low-temperature stress to maintain the integrity of the cell membrane, improve the antioxidant capacity (Parvanova et al., 2004; Arminian and Bidgoli, 2019), provide material energy for plant cells (Zhao et al., 2006), and thus increase the cold resistance of plants and reduce the damage caused by low temperature (Luo et al., 2004; Zhu et al., 2020; Sun et al., 2022). In this study, the contents of Pro, SS and SP all increased after low-temperature stress, the contents in OE plants were always significantly greater than those in WT and RNAi plants, and the accumulation at -5°C was greater than that at 4°C. The SS and SP contents of the RNAi plants were the same as those of the WT plants after low-temperature treatment but were still significantly lower than those of the OE plants. These results indicated that low temperature induced the accumulation of osmoregulatory substances to resist cold stress and that the response of plants to freezing injury was greater than that to cold injury.

The ICE-CBF-COR pathway is a key signal transduction pathway involved in the response of plants to low-temperature stress. As the hub of the cold resistance pathway, CBF binds to the CRT/DRE sequence in the promoter of the *COR* gene under low-temperature conditions and directly promotes transcription in response to low-temperature stress, increasing the ability to resist cold (Tian et al., 2023). Studies have shown that CCA1/LHY can bind to the CBF promoter to play a positive regulatory role (Seo et al., 2012; Nakamichi et al., 2016), and the cold-induced expression of *CBF* is significantly reduced in *Arabidopsis cca1 lhy* double mutants (Dong et al., 2011). Inhibition of *CsLHY* in tea plants decreases the expression of *CsCBF1*, which in turn decreases the cold tolerance of tea plants (Wu et al., 2024). Under 4°C and -5°C stress, the expression of *MsCBF1* in the OE plants was significantly induced and upregulated, whereas the expression in the RNAi plants was significantly lower than that in the WT and OE plants. The expression of *MsCOR15A* followed the same trend, indicating that *MsLHY* may mainly regulate downstream *MsCOR15A* by binding to *MsCBF1* to promote cold resistance in alfalfa.

## 5 Conclusion

We isolated and identified the *MsLHY* gene in alfalfa. The overexpression of *MsLHY* increases the activity of antioxidant enzymes and osmoregulatory substances, reduces the accumulation of reactive oxygen species, and improves the cold tolerance of alfalfa with less ion leakage. OE plants under low-temperature stress presented increased expression of cold tolerance genes and antioxidase-encoding genes and increased cold tolerance. In conclusion, *MsLHY* is an important candidate gene for the development of cold-resistant alfalfa varieties.

## Data Availability

The data presented in the study are included in the article/**Supplementary Material**.
