# Supplementary material for: MsLHY is an active regulator of cold resistance in alfalfa (Medicago sativa L.)
Source: Front Plant Sci. 2025 May 1;16:1559988. doi: 10.3389/fpls.2025.1559988 (PMC12078331; doi:10.3389/fpls.2025.1559988)
Supplement: Supplementary file 1 [file DataSheet1.docx]

**Supplementary material**

Supplementary Table S1 List of primers used in this study.

| Primer name | Sequence (5' to 3') |
| --- | --- |
| *MsLHY*-F | ATGGATGCAGCAGCATACTCCT |
| *MsLHY*-R | TCAAATAGAAGTCCCCCCTTCC |
| PEG100-*MsLHY*-OE-F | ACTATTTACAATTACGGATCatgtcttctacattttcaagtcttattgtctctaccttg |
| PEG100-*MsLHY*-OE-R | GGTCTTAATTAACTCTCTAGtcaaatagaagtccccccttccagg |
| PEG100-*MsLHY*-RNAi-1-F | cagtGGTCTCagatcacagtagctgctgcaactgcatgg |
| PEG100-*MsLHY*-RNAi-1-R | cgatGGTCTCacaggttctggatctagtaactgatcttgcagagg |
| PEG100-*MsLHY*-RNAi-2-F | cgatGGTCTCacctgcaggtctagtttttctccttcattttc |
| PEG100-*MsLHY*-RNAi-2-R | cgatGGTCTCagcccgggctctgtaactatcatc |
| PEG100-*MsLHY*-RNAi-3-F | cagtGGTCTCagggcttctggatctagtaactgatcttgcagagg |
| PEG100-*MsLHY*-RNAi-3-R | cagtGGTCTCactagacagtagctgctgcaactgcatgg |
| *MsLHY*-qPCR-F | CTGTGCACGTTGTTGATGGG |
| *MsLHY*-qPCR-R | GCAGCTGGATTTGCGAAGAG |
| *MsICE1*-qPCR-F | AAGCTTCAGCAATGGCTACTTC |
| *MsICE1*-qPCR-R | GGGCAAGCTCAACTGAGTAGT |
| *MsCBF1*-qPCR-F | TGCCACTGAGGAGCAGAGTA |
| *MsCBF1*-qPCR-R | CAGGGGACATTAGCGCCATA |
| *MsCOR15A*-qPCR-F | AGTTAGAGCTGGCTTGGCTC |
| *MsCOR15A*-qPCR-R | TGGCGGAGTTTTGTCTCCAA |
| *MsSOD1*-qPCR-F | GATGGAACCGCAAGCTTCAC |
| *MsSOD1*-qPCR-R | GCTCATGACCACCTTTCCCA |
| *MsCAT1*-qPCR-F | ACTCCTCCCTAACCGTTGGA |
| *MsCAT1*-qPCR-R | ACACGTTCTGGGATCCGTTC |
| *MsCML10*-qPCR-F | TCAGCCAGTGAGTTGAGACAC |
| *MsCML10*-qPCR-F | CAACTTGACCATCACCATCCA |
| Bar-F | GCTGAAGTCCAGCTGCCAGA |
| Bar-R | CGCAACGCCTACGACTGGAC |
| *GAPDH-*F | GGCTGCCATCAAGGAGGAAT |
| *GAPDH-*R | TCCAAGCTCAGCCTCATCAAG |


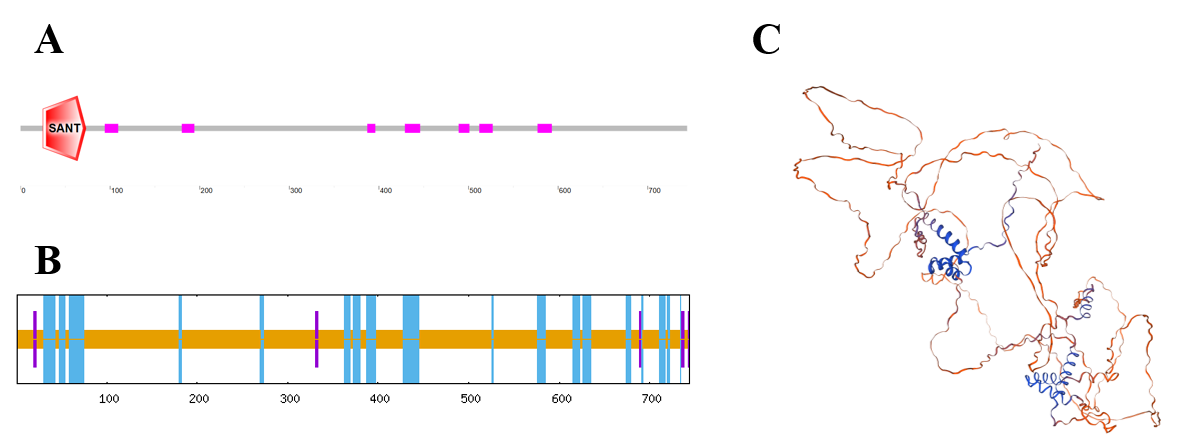


Figure S1 Prediction of the structure of the MsLHY protein: (A) prediction of the domains of the MsLHY protein; (B) prediction of the secondary structure of the MsLHY protein; (C) prediction of the tertiary structure of the MsLHY protein.


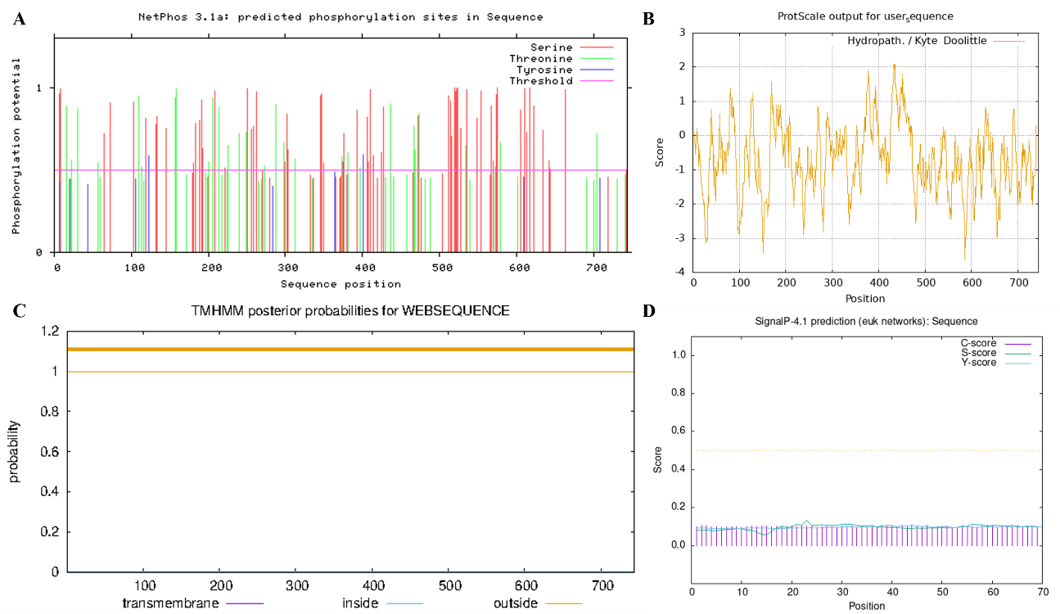


Figure S2 Prediction of MsLHY protein functions: (A) prediction of phosphorylation sites; (B) prediction of protein hydrophobicity; (C) prediction of transmembrane domains of the MsLHY protein; (D) prediction of protein signal peptides.


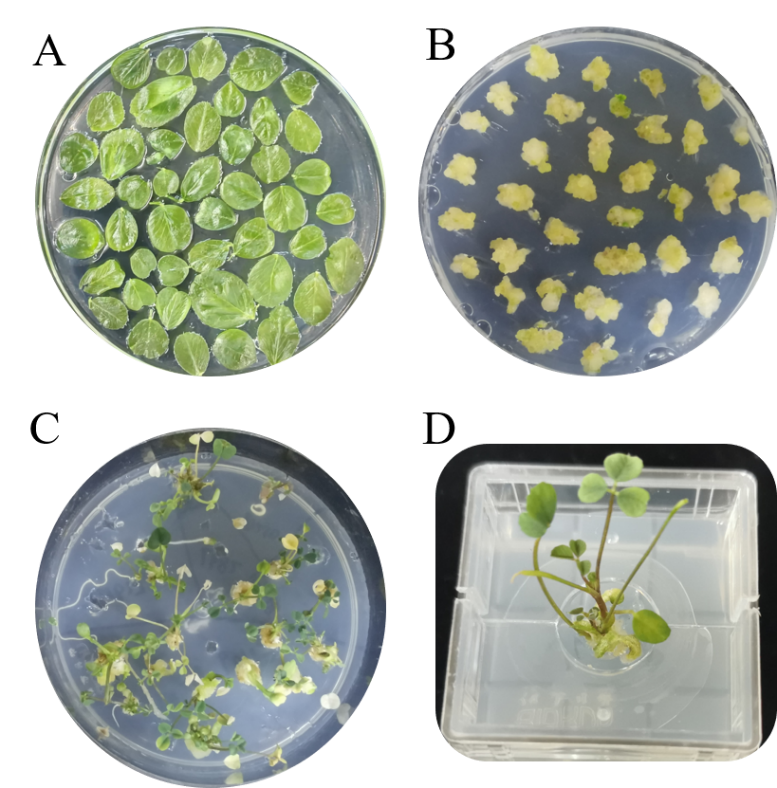


Figure S3: Transgenic plants: (A) preculture; (B) callus induction; (C) bud differentiation; (D) root induction.


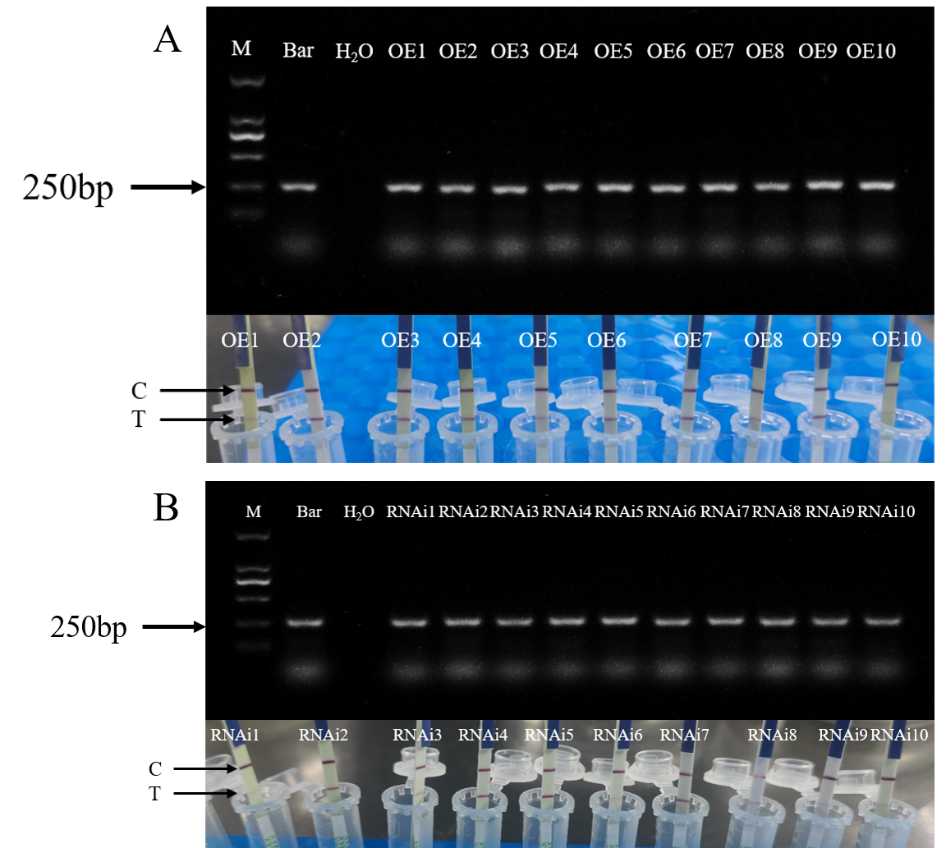


Figure S4: Positive detection of transgenic alfalfa, with a positive control of a 230 bp specific sequence of the Bar gene and a negative control of H_2_O; both the C and T lines of the Bar test strip appeared, indicating the presence of the Bar gene in the sample. (A) Positive detection of *MsLHY-*overexpressing plants; (B) positive detection of RNAi plants.


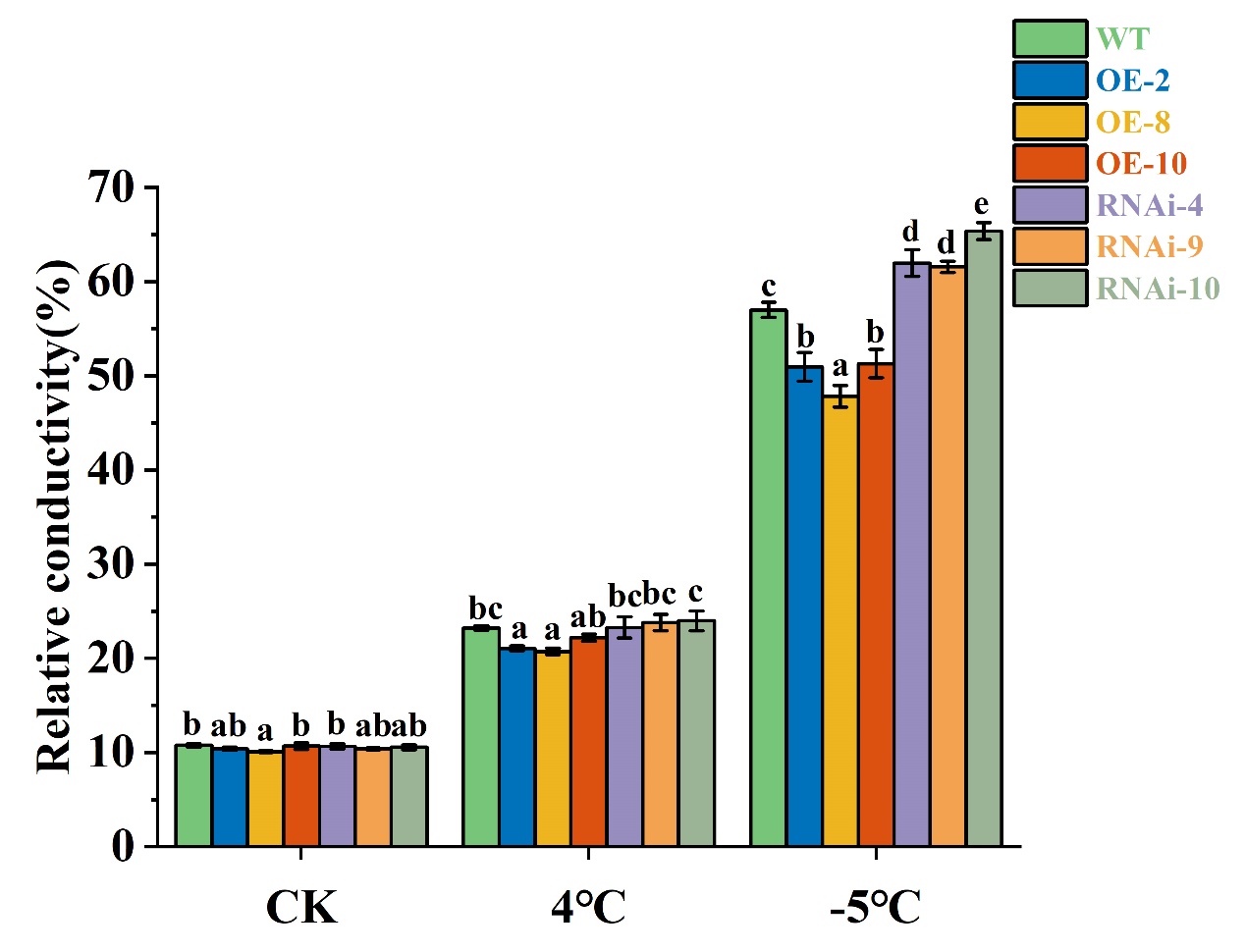


Figure S5: Relative conductivity of alfalfa after low temperature stress.
